# Technology access and preferences for remote assessments at Alzheimer's Disease Research Centers

**DOI:** 10.1002/alz.71467

**Published:** 2026-05-11

**Authors:** Carol K. Chan, Kathleen A. Lane, Sujuan Gao, Omolola A. Adeoye‐Olatunde, Patrick Shih, David K. Johnson, Andrew J. Saykin, Sophia Wang

**Affiliations:** ^1^ Center for Brain Health, Neurological Institute Cleveland Clinic Cleveland Ohio USA; ^2^ Department of Biostatistics and Health Data Science Indiana University School of Medicine Indianapolis Indiana USA; ^3^ Purdue University College of Pharmacy, Center for Health Excellence, Quality and Innovation Purdue University West Lafayette Indiana USA; ^4^ Luddy School of Informatics, Computing, and Engineering Indiana University Bloomington Bloomington Indiana USA; ^5^ Department of Neurology University of California Davis School of Medicine Walnut Creek California USA; ^6^ Indiana Alzheimer's Disease Research Center Indiana University School of Medicine Indianapolis Indiana USA; ^7^ Center for Neuroimaging, Department of Radiology and Imaging Sciences Indiana University School of Medicine Indianapolis Indiana USA; ^8^ Department of Psychiatry Indiana University School of Medicine Indianapolis Indiana USA

**Keywords:** access, Alzheimer's disease, race and ethnicity, technology

## Abstract

**BACKGROUND:**

As Alzheimer's disease and related dementias studies incorporate remote assessments, people at higher risk, such as individuals from minority racial and ethnic groups, may be disadvantaged due to imbalances in access.

**METHODS:**

Using data from the National Alzheimer's Coordinating Center Uniform Data Set, logistic regression models using generalized estimating equations with a random effect for study site were used to test the association of race and ethnicity, education, and their interactions with technology preferences and internet access.

**RESULTS:**

A total of 3,803 participants across 17 ADRCs (mean age 73.3 years [standard deviation [SD] = 9.9], education 16.4 years [SD = 2.7], 79% non‐Hispanic White) were included. The effect of education on internet access via desktop, laptop computer, and tablet was greater in non‐White participants than in non‐Hispanic White participants. A similar pattern was observed for interest in using devices for study visits.

**DISCUSSION:**

Education may have a role in racial and ethnic differences in technological access and preferences.

## Highlights

- The effect of higher education on internet access via desktop, laptop computer, and tablet was greater in non‐White participants than non‐Hispanic White participants.
- A similar pattern of results was observed for interest in using devices for study visits.
- Education may have a role in racial and ethnic differences in technological access and preferences for remote research assessments.
- Future studies should consider these differences when designing protocols with remote data collection.

## 1 BACKGROUND

During the COVID‐19 pandemic, Alzheimer's Disease Research Centers (ADRCs) adjusted their strategies to allow for virtual assessments,^1^ providing a roadmap of how technology may be utilized to facilitate remote study visits. The adoption of remote research visits and cognitive testing has advantages, particularly for longitudinal research programs that require long‐term participant commitments. They have the potential to lower participant burden and facilitate access to individuals who may otherwise be excluded due to long travel distances, transportation difficulties, and physical frailty. Investigators should, however, consider the accessibility of technology in the populations they are studying to avoid selection bias due to disparities in internet access, device ownership, and comfort with technology utilization.^2^

In the United States, older adults are increasingly using technology. The Pew Research Center reported that in 2021, 61% of older adults owned a smartphone, 44% owned a tablet, and only 25% reported never using the internet.^3^, ^4^ In spite of increased adoption, thier use of technology and the internet lags behind younger groups, and adoption has been primarily been driven by 65‐ to 69‐year‐olds with higher incomes and/or higher educational attainment.^5^ A survey of older adults receiving primary care services found that those without access to technology to enable video visits were more likely to be insured by Medicare, have limited English proficiency and a higher number of medical comorbidities.^6^ These usage trends may affect the types of patients that remote assessments can reach.

Beyond technological disparities across age, income, and education, racial and ethnic differences in technology use have also been reported. Black and Hispanic adults in the United States are less likely than non‐Hispanic White adults to have a desktop computer or broadband in the home.^7^ Older Black and Hispanic adults are less likely to have access to electronic devices that enable a video visit^6^ and are less likely to use technology for health‐related purposes^8^ compared to their non‐Hispanic White counterparts.

As more ADRD studies incorporate digital strategies and remote assessments, such disparities in digital access could further exacerbate research inequities for populations at higher risk of developing dementia. These populations at higher risk often include older adults who have less access to dementia specialist care based at tertiary academic medical centers due to population level disparities in health insurance and geographical access and often have comorbid medical and psychiatric disorders, which are usually exclusion criteria for research participation.^9^, ^10^ The National Institute on Aging (NIA) has identified fundamental factors of health disparities in their Health Disparities Research Framework, including but not limited to environmental, sociocultural, psychological, behavioral, and biological factors.^11^ Together, these factors can simultaneously contribute to a population's increased risk for ADRD and lower research participation, limiting the generalizability of research advances to populations of higher risk. Addressing the disproportionate under‐enrollment of populations at higher risk is essential to improving our understanding of heterogenous causal mechanisms, modifiable risks and protective factors, and responses to treatments and to finding solutions to reduce the public health burden of ADRD.

Race and ethnicity are proxies for many psychosocial factors related to an increased risk of dementia. To our knowledge, however, prior studies of US ADRD cohorts examining racial and ethnic differences in access to technology did not directly examine the potential interaction between these factors.^2^, ^12^

To better understand ways technological disparities could affect the participation of populations at higher risk in ADRD studies, these analyses compared patterns of technology access and preferences across racial and ethnic groups at NIA‐funded ADRCs in the United States. Using the National Alzheimer's Coordinating Center (NACC) Uniform Data Set (UDS) COVID‐19 Technology Accessibility Survey (CTAS), these analyses build on a prior study of the CTAS that utilized data collected until November 2021^12^ by providing updated analyses with a larger cohort and examining the relationship between race and ethnicity and education. Specifically, our objectives were to (1) compare patterns of technology access and study visit preferences across racial and ethnic groups and (2) examine the relationship between education and race and ethnicity on patterns of technology access and study visit preferences.

## 2 METHODS

### 2.1 National Alzheimer's Coordinating Center Uniform Data Set

Data for this secondary analysis were obtained from the NACC UDS Version 3.0.^13^ The NACC database includes standardized research data collected from NIA‐funded ADRCs across the United States and reflects the total enrollment of ADRCs since 2005.

Data collection was approved by the Institutional Review Board at each participating ADRC. Written informed consent was obtained from all participants and co‐participants. Recruitment and participant evaluation are described in detail elsewhere.^13^ Briefly, participants were enrolled by each ADRC according to their own protocol and criteria. Data for the UDS were collected using standardized evaluation forms. Participants across the cognitive spectrum were included in the NACC UDS, including those with normal cognition, mild cognitive impairment (MCI), and dementia.

### 2.2 Covid‐19 technology access survey

During the COVID‐19 pandemic, versions of the standardized NACC assessment were developed for remote administration, either by telephone or video conferencing. As part of the change from in‐person to remote assessments, a survey (the COVID‐19 Technology Accessibility Survey [CTAS]) was developed to better understand the preferences and technological capabilities of study participants. The survey included the following questions: (1) “How would you prefer to conduct a study visit with us?” Responses (select your top choice): In person, telephone call, video call, no preference, or decline to answer. (2) “How do you currently access the internet?” Responses (check all that apply): smartphone, tablet/iPad, laptop, desktop computer, or other. (3) “Do you use email to receive and send documents?” Responses: no, yes, or decline to answer. (4) “Are you interested in using any of the following to do some parts of your study visit at home?” Responses (check all that apply): smartphone, tablet/iPad, laptop, desktop computer, wearable device (e.g., FitBit, Applewatch), smart home devices (e.g., Xbox, Nest), or other. Question 3, concerning the use of email to receive and send documents, was excluded from these analyses as it was less relevant to the aims of the study. The survey was provided to all ADRCs starting on July 2, 2020. Administration of the survey was optional for ADRCs. Within ADRCs that offered the survey, its completion was optional for participants. Participants were allowed to skip any of the questions on the questionnaire. As a result, the data collected represented a convenience sample. The survey was completed either by the participant (if Clinical Dementia Rating [CDR] Dementia Staging instrument score = 0 or 0.5) or by the co‐participant/caregiver on behalf of the research participant (if CDR score > 0.5). Ten out of 17 sites collected responses to the CTAS multiple times. For participants who completed the survey multiple times, only the most recent responses were used. Four participants had responses to Questions 2 and 4 documented as “missing” despite having answered “yes” to other options of the same question. These responses were therefore changed from “missing” to “no” for the analyses.

**RESEARCH IN CONTEXT**

1. **Systematic review**: The authors reviewed the literature using traditional sources. There have been longstanding challenges in the recruitment and retention of populations at higher risk of dementia for ADRD studies. This includes but is not limited to participants with fewer years of education and people who do not identify as non‐Hispanic White, both of which are associated with a higher risk of ADRD and disproportionately low participation in ADRD studies.
2. **Interpretation**: Education may have a role in racial and ethnic differences in technological access and preferences for remote research assessments.
3. **Future directions**: Future studies should account for preferences for assessment modalities and technologies used when designing protocols. Special consideration should be given to the accessibility of technology and willingness of participants to participate in remote research visits as these factors may affect the recruitment and retention of populations at higher risk of dementia.

### 2.3 Participant selection

These analyses considered all participants enrolled in the NACC UDS who had completed the CTAS between July 2, 2020, and April 26, 2023.

### 2.4 Demographic, cognitive, and clinical variables

Demographic, cognitive, and clinical variable data were extracted from participants’ visit records when the CTAS was completed. Race (categorized in the NACC UDS as White, Black or African American, Asian, American Indian or Alaska Native, Native Hawaiian or Other Pacific Islander, multiracial, or unknown) and ethnicity (Hispanic or Latino, non‐Hispanic) were determined by self‐report from participants’ initial visit record. Five racial ethnic groups were created: non‐Hispanic White, Hispanic, non‐Hispanic Black, non‐Hispanic Asian, and Other race. Participants who were classified as multi‐racial and selected Black or African American were included as non‐Hispanic Black. Non‐Hispanic Asians were included similarly. “Other” race included American Indian or Alaska Native, Native Hawaiian or Other Pacific Islander, and remaining multi‐racial participants who did not also identify as non‐Hispanic Black or non‐Hispanic Asian.

Criteria for cognitive status classification are detailed elsewhere.^13^, ^14^ The CDR^15^ score is derived from clinician rating of cognition and function, where scores of 0, 0.5, 1, 2, and 3 correspond to no impairment, MCI, mild dementia, moderate dementia, and severe dementia, respectively.

### 2.5 Statistical analysis

Analyses were conducted in two ways. First, we combined all participants who did not identify as non‐Hispanic White into the “non‐White” group and compared the with the non‐Hispanic White group. The analysis was then repeated using four racial and ethnic categories: Hispanic, non‐Hispanic White, non‐Hispanic Black, and non‐Hispanic Asian and other. Non‐Hispanic Asian and other were combined due to small sample sizes in each category (*n* = 65 and *n* = 53, respectively). Non‐Hispanic White was the reference group in all analyses.

Descriptive statistics including means and standard deviations for continuous variables and count and percentage for categorical variables were calculated between the racial/ethnic groups on patient characteristics and survey data. *T*‐tests, one‐way ANOVA models, and chi‐squared tests were used to compare the racial/ethnic groups on these data as appropriate. Multinomial logistic regression models using generalized estimating equations (GEEs) to adjust for study site were used to compare study modality. Using in‐person visits as the reference group, we examined preference for telephone, video, and no preference for study visit modality.

Independent variables in the multinomial logistic model included race and ethnicity, education, and the interaction between education and race and ethnicity while adjusting for age, sex, and CDR. For Item 2 (devices used to access the internet), four separate logistic regression models using GEE to adjust for study site were conducted to evaluate the odds of selecting versus not selecting each device type (smartphone, tablet, laptop, desktop computer) as separate outcomes. For Item 4 (interest in using devices to complete study visits at home), six separate logistic regression models using GEEs to adjust for study site were conducted to evaluate the odds of selecting versus not selecting each device type (smartphone, tablet, laptop, desktop computer, wearable device, smart home device). Predictor variables in all models included race/ethnicity group, years of education, the race/ethnicity group × years of education interaction, and covariates of age, sex, and global CDR. Because responses to these items were “check all that apply,” each device was analyzed separately. If participants selected a device, their response was coded as “yes.” If they did not select a device, their response was coded as “no.” Race and ethnicity, education, and the interaction between education and race and ethnicity were the major independent variables adjusting for age, sex, and CDR. Statistical analyses were completed using SAS version 9.4. Significance was set at alpha = 0.05.

### 2.6 Selection bias analysis

Because the administration and completion of the CTAS was optional, we sought to examine whether there were differences between participants who completed the survey versus those who did not. We created four groups: (1) participants who completed CTAS; (2) participants who had a visit on/after July, 2, 2020, through April 23, 2023, but not did not complete CTAS at sites that administered CTAS; (3) participants who had a visit during the time period of interest at sites that did not administer CTAS; and (4) participants with visits in the 3 years prior to the CTAS becoming available (July 2, 2020), but no visits afterwards, as a proxy for participant dropout during the COVID‐19 pandemic. The 3‐year window was chosen based on the definition of attrition in a prior NACC analysis (Burke et al., 2019). One‐way ANOVA and chi‐squared tests were used to compare the groups on continuous and categorical characteristics, respectively. Additional unadjusted pairwise chi‐squared tests were used to test each participation group against Group 1 (those who had completed the survey) as this was the reference group.

## 3 RESULTS

The primary results using two groups (non‐Hispanic White and non‐White participants) are shown in Tables 1, 2, 3, 4. Results from analyses using four racial and ethnic categories (non‐Hispanic White, non‐Hispanic Black, non‐Hispanic Asian and other, Hispanic) are included in Tables S1–S4.

**TABLE 1:** Participant characteristics. CDR, clinical dementia rating scale; yrs, years; MCI, mild cognitive impairment; SD, standard deviation.

	Non‐Hispanic White		Non‐White		
Variable	*N*	Statistic	*N*	Statistic	Overall *p* value
Age (years) at visit, mean ± SD	3022	73.3 ± 10.1	781	73.3 ± 9.3	0.880
Female, *n* (%)	3022	1606 (53.1%)	781	557 (71.3%)	<0.001
Years of education, mean ± SD	3012	16.6 ± 2.5	781	15.4 ± 3.2	<0.001
Cognitive status at UDS visit, *n* (%)	3022		781		<0.001
Normal cognition		1750 (57.9%)		513 (65.7%)	
Impaired – not MCI		159 (5.3%)		33 (4.2%)	
MCI		448 (14.8%)		149 (19.1%)	
Dementia		665 (22.0%)		86 (11.0%)	
Global CDR, *n* (%)	3022		781		<0.001
0.0		1653 (54.7%)		464 (59.4%)	
0.5		885 (29.3%)		255 (32.7%)	
≥1		484 (16.0%)		62 (7.9%)	
Indicator of first‐degree family member with cognitive impairment, *n* (%)	2846	1781 (62.6%)	709	387 (54.6%)	<0.001
Principal referral source, *n* (%)	2998		775		<0.001
Non‐professional contact		1115 (37.2%)		318 (41.0%)	
Professional contact		1338 (44.6%)		260 (33.5%)	
Other referral		545 (18.2%)		197 (25.4%)	
Preference to conduct a study visit, *n* (%)	2944		759		0.108
In person		1280 (43.5%)		320 (42.2%)	
Telephone call		523 (17.8%)		162 (21.3%)	
Video call		530 (18.0%)		120 (15.8%)	
No preference		611 (20.8%)		157 (20.7%)	
Current access to internet via	3022		781		
Smartphone, *n* (%)		2076 (68.7%)		517 (66.2%)	0.181
Tablet/iPad, *n* (%)		1289 (42.7%)		309 (39.6%)	0.119
Laptop, *n* (%)		1838 (60.8%)		429 (54.9%)	0.003
Desktop computer, *n* (%)		1280 (42.4%)		299 (38.3%)	0.040
Uses email to receive and send documents, *n* (%)	2978	2620 (88.0%)	761	615 (80.8%)	<0.001
Interested in using device for study visits	3022		781		
Smartphone, *n* (%)		1675 (55.4%)		440 (56.3%)	0.648
Tablet/iPad, *n* (%)		1177 (38.9%)		303 (38.8%)	0.938
Laptop, *n* (%)		1694 (56.1%)		401 (51.3%)	0.018
Desktop computer, *n* (%)		1130 (37.4%)		285 (36.5%)	0.642
Wearable devices, *n* (%)		12 (20.3%)		149 (19.1%)	0.465
Smart home devices, *n* (%)		258 (8.5%)		68 (8.7%)	0.880

*Note*: Ten participants had missing data for education; 248 had missing first‐degree family member with cognitive impairment; 742 had missing data for principal referral source. For preference to conduct study visit, 98 participants declined to answer, two had missing data. For email use, 64 declined to answer.

**TABLE 2:** Multinomial logistic regression model using generalized estimating equations with random effect for study site on study visit modality preferences in non‐White and non‐Hispanic White participants.

	Telephone versus in person			Video versus in person			No preference versus in person		
Independent variable	OR	95% CI	*p*‐value	OR	95% CI	*p*‐value	OR	95% CI	*p*‐value
Age (years) at visit	1.02	1.00 to 1.01	0.007	0.99	0.97 to 1.00	0.134	0.99	0.97 to 1.00	0.023
Female	1.40	1.14 to 1.72	<0.001	1.23	1.06 to 1.44	0.008	1.13	0.83 to 1.53	0.429
Years of education	0.97	0.92 to 1.02	0.268	1.08	1.04 to 1.12	<0.001	1.04	1.00 to 1.08	0.061
Global CDR									
0.0	0.63	0.27 to 1.50	0.298	0.59	0.25 to 1.40	0.227	1.27	0.66 to 2.43	0.479
0.5	0.61	0.26 to 1.45	0.260	0.59	0.29 to 1.19	0.140	0.87	0.56 to 1.35	0.525
≥1	1.00	1.00 to 1.00		1.00	1.00 to 1.00		1.00	1.00 to 1.00	
Non‐White	0.90	0.35 to 2.32	0.830	0.23	0.04 to 1.40	0.111	1.58	0.56 to 4.47	0.391
Non‐Hispanic White	1.00	1.00 to 1.00		1.00	1.00 to 1.00		1.00	1.00 to 1.00	
Education × Non‐White	1.02	0.97 to 1.07	0.518	1.09	0.98 to 1.23	0.118	0.97	0.92 to 1.04	0.405
Education × Non‐Hispanic White	1.00	1.00 to 1.00		1.00	1.00 to 1.00		1.00	1.00 to 1.00	

*Note*: Adjusted for age, sex, education, CDR, and race‐education interaction. Reference group, in‐person visit.

Abbreviations: CDR, Clinical Dementia Rating scale; CI, confidence interval; OR, odds ratio.

**TABLE 3:** Separate logistic regression models using generalized estimating equations with random effect for study site on current devices used to access the internet in non‐White and non‐Hispanic White participants.

	Smartphone			Tablet			Laptop			Desktop		
Independent variable	OR	95% CI	*p* value	OR	95% CI	*p* value	OR	95% CI	*p* value	OR	95% CI	*p* value
Age (years) at visit	0.93	0.92 to 0.94	<0.001	0.98	0.97 to 0.99	<0.001	0.95	0.94 to 0.96	<0.001	1.00	0.99 to 1.01	0.978
Female	0.82	0.71 to 0.94	0.005	1.07	0.95 to 1.20	0.290	0.87	0.75 to 1.00	0.048	0.81	0.74 to 0.88	<0.001
Years of education	1.06	1.02 to 1.10	0.004	1.03	1.00 to 1.06	0.033	1.11	1.07 to 1.15	<0.001	1.04	1.01 to 1.07	0.021
Global CDR – overall			<0.001			0.051			0.005			0.112
0.0	1.45	0.93 to 2.26	0.104	1.31	1.01 to 1.71	0.044	1.79	1.25 to 2.55	0.001	1.31	1.01 to 1.71	0.040
0.5	0.98	0.67 to 1.43	0.909	1.06	0.86 to 1.32	0.575	1.33	1.06 to 1.68	0.016	1.18	0.91 to 1.54	0.210
≥1	1.00	1.00 to 1.00		1.00	1.00 to 1.00		1.00	1.00 to 1.00		1.00	1.00 to 1.00	
Non‐White	1.23	0.40 to 3.86	0.717	0.32	0.17 to 0.59	<0.001	0.14	0.04 to 0.51	0.003	0.36	0.15 to 0.87	0.024
Non‐Hispanic White	1.00	1.00 to 1.00		1.00	1.00 to 1.00		1.00	1.00 to 1.00		1.00	1.00 to 1.00	
Education × Non‐White	0.98	0.91 to 1.05	0.565	1.07	1.03 to 1.10	<0.001	1.12	1.04 to 1.22	0.004	1.06	1.01 to 1.12	0.023
Education × Non‐Hispanic White	1.00	1.00 to 1.00		1.00	1.00 to 1.00		1.00	1.00 to 1.00		1.00	1.00 to 1.00	

*Note*: Adjusted for age, sex, education, CDR, and race‐education interaction.

Abbreviations: CDR, Clinical Dementia Rating scale; CI, confidence interval; OR, odds ratio.

**TABLE 4:** Separate logistic regression models using generalized estimating equations with random effect for study site on interest in using devices to complete parts of their study visits in non‐White and non‐Hispanic White participants.

	Smartphone			Tablet			Laptop			Desktop			Wearable device			Smart home device		
Independent variable	OR	95% CI	*p* value	OR	95% CI	*p* value	OR	95% CI	*p* value	OR	95% CI	*p* value	OR	95% CI	*p* value	OR	95% CI	*p* value
Age (years) at visit	0.95	0.94 to 0.97	<0.001	0.97	0.96 to 0.98	<0.001	0.95	0.94 to 0.96	<0.001	0.99	0.98 to 1.00	0.123	0.96	0.95 to 0.97	<0.001	0.96	0.95 to 0.97	<0.001
Female	0.87	0.80 to 0.95	0.002	1.07	0.96 to 1.20	0.218	0.87	0.73 to 1.02	0.089	0.82	0.75 to 0.91	<0.001	1.06	0.89 to 1.26	0.538	0.92	0.70 to 1.21	0.554
Years of education	1.05	1.02 to 1.08	<0.001	1.04	1.01 to 1.08	0.013	1.12	1.08 to 1.17	<0.001	1.04	1.01 to 1.06	0.002	1.06	1.04 to 1.10	<0.001	1.08	1.04 to 1.12	<0.001
Global CDR –overall			0.027			0.065			<0.001			0.078			0.041			0.870
0.0	1.21	0.85 to 1.71	0.288	1.27	1.04 to 1.56	0.020	1.85	1.40 to 2.46	<0.001	1.35	1.03 to 1.77	0.028	1.50	1.09 to 2.06	0.013	1.08	0.72 to 1.60	0.713
0.5	0.92	0.71 to 1.19	0.510	1.15	0.97 to 1.37	0.114	1.30	1.08 to 1.57	0.005	1.23	0.99 to 1.53	0.060	1.30	0.95 to 1.80	0.105	1.12	0.73 to 1.71	0.601
≥1	1.00	1.00 to 1.00		1.00	1.00 to 1.00		1.00	1.00 to 1.00		1.00	1.00 to 1.00		1.00	1.00 to 1.00		1.00	1.00 to 1.00	
Non‐White	1.45	0.36 to 5.88	0.603	0.32	0.20 to 0.54	<0.001	0.18	0.06 to 0.55	0.003	0.38	0.17 to 0.89	0.025	0.39	0.15 to 1.01	0.053	0.45	0.13 to 1.63	0.224
Non‐Hispanic White	1.00	1.00 to 1.00		1.00	1.00 to 1.00		1.00	1.00 to 1.00		1.00	1.00 to 1.00		1.00	1.00 to 1.00		1.00	1.00 to 1.00	
Education × Non‐White	0.98	0.90 to 1.07	0.593	1.07	1.03 to 1.10	<0.001	1.11	1.03 to 1.18	0.003	1.06	1.01 to 1.11	0.015	1.05	0.99 to 1.11	0.086	1.05	0.97 to 1.13	0.260
Education × Non‐Hispanic White	1.00	1.00 to 1.00		1.00	1.00 to 1.00		1.00	1.00 to 1.00		1.00	1.00 to 1.00		1.00	1.00 to 1.00		1.00	1.00 to 1.00	

*Note*: Adjusted for age, sex, education, CDR, and race‐education interaction.

Abbreviations: CI, confidence interval; CDR, Clinical Dementia Rating scale; OR, odds ratio.

### 3.1 Participant characteristics

These analyses included data from 3,803 participants across 17 ADRCs (Table 1), representing approximately 25% of the total active participants in the NACC database during the same period. It included 781 (21%) individuals who identified as non‐White and 3,022 (79%) non‐Hispanic White participants.

Compared to non‐Hispanic White, the non‐White group had a higher percentage of female participants, lower education, less cognitive impairment, and fewer participants with a first‐degree family member with cognitive impairment.

### 3.2 Study visit modality preferences

In non‐Hispanic White participants, 1280 (43.5%) selected in‐person visit as their top choice for study visit modality, while 523 (17.8%) selected telephone call, 530 (18.0%) selected video call, and 611 (20.8%) reported no preference (Table 1). In non‐White participants, 320 (42.4%) selected in‐person visit as their top choice for study visit modality, while 162 (12.3%) selected telephone call, 120 (15.8%) selected video call, and 157 (20.7%) reported no preference (Table 1). No significant differences were observed between non‐Hispanic White and non‐White groups.

There were no significant interactions between race and ethnicity and education for study visit modality preference (Table 2). Further, there were no differences between non‐White and non‐Hispanic White participants’ preferences for study visit modality. However, female and more educated participants preferred video visit over in‐person visits (Female: OR = 1.23, 95% CI = 1.06 to 1.44, *p *= 0.008; education: OR = 1.08, 95% CI = 1.04 to 1.12, *p *< 0.001). Female and older participants had higher odds of preferring a telephone visit over an in‐person visit (Female: OR = 1.40, 95% CI = 1.14 to 1.72, *p *< 0.001; age: OR = 1.02, 95% CI = 1.00 to 1.01, *p* = 0.007). When race/ethnicity was analyzed across four groups, there were again no significant interactions between race or ethnicity with education for study visit modality (Table S2). No significant differences across race and ethnicity were observed. Participants with more years of education had higher odds of preferring a video visit over an in‐person visit (OR = 1.08, 95% CI = 1.04 to 1.12, *p *< 0.001).

### 3.3 Current device used to access the internet

In non‐Hispanic White participants, 2,076 (68.7%), 1289 (42.7%), 1,838 (60.8%), and 1280 (42.4%) reported current access to the internet via smartphone, tablet/iPad, laptop, and desktop computer, respectively (Table 1). In non‐White participants, 517 (66.2%), 309 (39.6%), 429 (54.9%), and 299 (38.3%) reported current access to the internet via smartphone, tablet/iPad, laptop, and desktop computer, respectively (Table 1). Significant differences between non‐Hispanic White and non‐White participants were observed for current access to internet via laptop and desktop computer.

There was a significant interaction between race and ethnicity and education for access to tablet, laptop, and desktop computer, where the effect of higher education was greater in non‐White participants than non‐Hispanic White participants (Table 3; Tablet: OR = 1.07, 95% CI = 1.03 to 1.10, *p* < 0.001; laptop: OR = 1.12, 95% CI = 1.04 to 1.22, *p* = 0.004; desktop: OR = 1.06, 95% CI = 1.01–1.12, *p* = 0.023). For current smartphone access, the interaction between race and ethnicity and education was not significant, and no significant differences across race and ethnicity were observed. However, more years of education were associated with higher odds of having current internet access via smartphone compared to fewer years of education (OR = 1.06, 95% CI = 1.02 to 1.20, *p *= 0.004). Compared to male participants, female participants had significantly lower odds of having access to the internet via smartphone and desktop computer, and older participants had significantly lower odds compared to younger participants of having access to the internet via smartphone, tablet, or laptop computer. Participants who were less cognitively impaired had higher odds of having access via tablet, laptop, or desktop computer compared to participants with greater cognitive impairment.

When analyzed as four racial and ethnic groups (Table S3), there was a significant interaction between education and racial or ethnic group overall for tablet and desktop access (*p *= 0.003 and *p *< 0.001, respectively). Hispanic participants with higher education had higher odds of tablet and desktop access compared to Non‐Hispanic White participants with less education (tablet: OR = 1.16, 95% CI = 1.05 to 1.29, *p* = 0.004; desktop: OR = 1.18, 95% CI = 1.08 to 1.30, *p* < 0.001). No significant interaction was observed overall between education and racial or ethnic groups for smartphone and laptop access. Higher education was associated with higher odds of current smartphone and laptop access (smartphone: OR = 1.06, 95% CI = 1.02 to 1.10, *p *= 0.003; laptop: OR = 1.11, 95% CI = 1.07 to 1.15, *p *< 0.001).

### 3.4 Interest in using devices for study visits

In non‐Hispanic White participants, 1,675 (55.4%), 1,177 (38.9%), 1,694 (56.1%), 1,130 (37.4%), 612 (20.3%), and 258 (8.5%) reported an interest in using a smartphone, tablet/iPad, laptop, desktop computer, wearable devices, and smart home devices, respectively (Table 1). In non‐White participants, 440 (56.3%), 303 (38.8%), 401 (51.3%), 285 (36.5%), 149 (19.1%), and 68 (8.7%) reported an interest in using a smartphone, tablet/iPad, laptop, desktop computer, wearable devices, and smart home devices, respectively (Table 1). A significant difference between non‐Hispanic White and non‐White participants was observed regarding an interest in using a laptop computer for study visits.

There was a significant interaction between education and racial or ethnic group in using a tablet, laptop, or desktop computer, where the effect of higher education on the interest was greater in participants who identified as non‐White than non‐Hispanic White participants (Table 4; tablet: OR = 1.07, 95% CI = 1.03 to 1.10, *p* < 0.001; laptop: OR = 1.11, 95% CI = 1.03 to 1.18, *p* < 0.003; desktop: OR = 1.06, 95% CI = 1.01 to 1.11, *p* = 0.015). No significant interaction was observed between education and racial or ethnic group in using a smartphone, wearable device, or smart home device. No differences were observed across racial and ethnic groups. However, higher education was associated with greater odds of interest in the use of these devices compared to participants with lower education (smartphone: OR = 1.05, 95% CI 1.02 to 1.08, *p* < 0.001; wearable device: OR = 1.06, 95% CI = 1.04 to 1.10, *p* < 0.001; smart home device: OR = 1.08, 95% CI = 1.04 to 1.12, *p* < 0.001).

When analyzed as four racial and ethnic groups (Table S4), no significant associations were observed in the interaction between education and individual racial and ethnic group overall for interest in using devices for study visits, except for tablet and laptop use (*p *< 0.0.01 and *p* = 0.006, respectively). Interactions between education and individual racial and ethnic groups were not significant for tablet use. A significant interaction between education and Hispanic ethnicity was observed for desktop use (OR = 1.17, 95% CI = 1.05 to 1.29, *p* = 0.003). Higher education was associated with higher odds of interest in using devices for study visits for smartphone, desktop computer, wearable device, and smart home device (smartphone: OR = 1.05, 95% CI = 1.02 to 1.08, *p *< 0.001; desktop: OR = 1.04, 95% CI = 1.01 to 1.06, *p* = 0.002; wearable device: OR = 1.07, 95% CI = 1.04 to 1.10, *p* < 0.001; smart home device: OR = 1.08, 95% CI = 1.04 to 1.12, *p* < 0.001).

### 3.5 Selection bias

Table S5 shows comparisons across (1) participants who completed CTAS; (2) participants who had a visit on/after July 2, 2020, through April 23, 2023, but not did not complete CTAS at sites that administered CTAS; (3) participants who had a visit during the time period of interest at sites that did not administer CTAS; and (4) participants with visits in the 3 years prior to the CTAS becoming available but no visits afterwards, as a proxy for participant dropout during the COVID‐19 pandemic. Significant differences were observed across groups in age, years of education, global CDR, and racial and ethnic groups. Compared to participants in Groups 2, 3, and 4, participants who completed the CTAS had more years of education, were less cognitively impaired, and were more likely to identify as non‐Hispanic White.

## 4 DISCUSSION

In a technology access survey, a higher percentage of non‐Hispanic White participants reported current access to the internet via tablet/iPad or laptop computer and an interest in using a laptop for study visits compared to non‐White participants. Non‐White participants and participants with fewer years of education enrolled in Alzheimer's research cohorts were less likely to access the internet using tablet, laptop, or desktop computer and were less interested in using these devices for parts of study visits. Significant interactions between race and education were observed across access to and interest in using a tablet, laptop, or and desktop, suggesting that education had a role in racial and ethnic differences in technological access and preferences. No differences were observed in non‐White participants versus non‐Hispanic White participants in study visit modality preferences. Participants who had more years of education preferred video visits over in‐person visits.

We did not observe significant racial or ethnic differences in current access to the internet via smartphone or interest in using a smartphone for study visits. This is consistent with prior reports showing that, while Black and Hispanic adults are less likely to have home broadband, smartphone ownership rates are high across all groups, with roughly 94% of Black and Hispanic internet users accessing the internet via mobile device.^16^ Remote study visit platforms configured for smartphone use may be a more accessible option than ones that require a tablet, laptop, or desktop computer. Mobile‐first approaches to designing web‐based recruitment registries to target recruitment of individuals from underrepresented communities have also been proposed as a way to improve enrollment.^17^

Though no racial or ethnic differences or interaction between race and education were observed, we did find that higher education was associated with greater odds of using smartphones and other devices to access the internet, pointing to education‐based disparities in device ownership and internet access. While the survey provides insight into the types of devices participants are using or have an interest in using, a limitation of the current survey due to its design and its voluntary nature is that it does not provide insight into participants who lack access to the internet entirely or have no interest in incorporating devices for study visits. A study using data from the 2018 National Health and Aging Trends Study, a longitudinal survey study of Medicare beneficiaries, found that non‐Hispanic White female participants, Black male participants, Black female participants, Hispanic male participants, and Hispanic female participants were all less likely to have a working cellphone or a working computer compared to non‐Hispanic White male participants.^18^ They similarly observed that participants who identified as non‐Hispanic White and had more years of education were more likely to have access to a working computer and cellphone. Other factors associated with higher likelihood of having a working computer included higher income and living in a metropolitan area. Participants who lack access to the internet entirely or have no interest in incorporating devices for study visits include groups at higher risk of ADRD, and further research is needed to examine how best to include such groups as studies move toward remote data collection and decentralized designs.

In older adults overall, including those without cognitive impairment, most do not feel confident in their ability to learn about and properly use electronic devices,^5^ with only 26% of older adults surveyed by the Pew Research Center reporting that they felt very confident in their ability to use computers, smartphones, or other electric devices to complete tasks online. Physical challenges such as limitations in vision, hearing, and dexterity may pose further challenges in connection with device use. These barriers may extend to difficulties in completing remote assessments, which usually require the use of devices connected to the internet. A study of older adults in independent living facilities found that top barriers to remote visits included not being familiar with the technology, having difficulty hearing, and a lack of interest in seeing providers outside of the clinic.^19^ Training programs for older adults with the aim of overcoming barriers related to unfamiliarity with technology have been studied and may provide a pathway for overcoming these barriers for interested participants.^6^, ^20^, ^21^

Our findings build on an earlier analysis of the CTAS using data collected up to November 15, 2021,^1^ representing approximately 12% (*n* = 2070) of the total active participants in the NACC database during the same period. In contrast to our findings, the authors observed that education and race were not associated with remote visit preferences, though they compared telephone or video call versus in‐person or no preference instead of comparing each option against in‐person visits. Education was associated with greater odds of interest in laptop use, but not smartphone, tablet, or desktop use, in contrast to our finding that it was associated with greater odds of interest across all devices. Possible reasons for contrasting results include differences in statistical analysis methods and the period when the survey was conducted – at the height of the COVID‐19 pandemic. This may have affected responses, particularly with visit modality preferences and comfort with technology. Technology adaptation evolved as the pandemic progressed, and more older adults adopted technological use in their day‐to‐day life out of necessity.^22^ Similar to our findings, though, the authors observed that cognitive impairment was associated with less interest in smartphone, tablet, and laptop use for study visits. They did not examine the association between race, ethnicity, or education on current device access.

Another technological use survey on an ADRC cohort conducted at the University of California San Diego^2^ between April 28, 2020, and June 8, 2020, found that while Hispanic participants were willing to do parts of their visit via video chat, they were less likely than non‐Hispanic participants to have high‐speed internet or device access. A similar pattern of results was observed for participants with lower educational attainment. Device access was higher in participants without dementia, and willingness to complete cognitive testing via video or smartphone was higher among participants without dementia compared to those with dementia.

The main strength of this study was the use of data from a large, nationwide database of well‐characterized participants. Our findings should, however, be interpreted within the context of their limitations. The survey was primarily completed during the height of the COVID‐19 pandemic, during which ADRCs each had their own site‐specific guidelines for research studies. The CTAS was voluntarily implemented by 17 out of 35 NIA‐funded ADRCs, and completion by participants at these ADRCs was voluntary. Thus, the data may be vulnerable to selection bias. In our analysis for selection bias, we observed that participants who completed the CTAS had higher education, were less cognitively impaired, and were more likely to identify as non‐Hispanic White than those who did not complete it at a participating site. This trend was also observed when comparing participants who completed the CTAS to participants from sites that did not participate in the CTAS, and participants who did not have any visits after the release of CTAS. We were also not able to adjust for rural versus urban settings due to limitations with data availability. The sample overall had a high level of education, with a mean of 16.4 (SD = 2.7) years of education. Due to certain racial and ethnic groups who had smaller numbers who completed the COVID‐19 survey, future studies will need to replicate these findings in cohorts with large numbers of these groups.

## 5 CONCLUSIONS

Our findings bring to light challenges that studies may face when implementing remote assessments and evidence that racial and ethnic differences in technology access in ADRC study participants may be in part driven by educational history. As the field moves toward decentralized clinical trials, future studies should account for preferences for assessment modalities and technologies used when designing protocols. While there are benefits to using remote data collection, investigators should consider the accessibility of technology and the willingness of participants to take part in remote research visits. Remote study platforms configured for smartphone use may provide a more accessible option than ones that require a tablet, laptop, or desktop computer. Special consideration should be given to how this may affect recruitment and retention of populations at higher risk of dementia, including but not limited to participants with fewer years of education and people who do not identify as non‐Hispanic White, both of which are associated with higher risk of ADRD and disproportionately low participation in ADRD studies.^23^

## CONFLICT OF INTEREST STATEMENT

The authors declare no conflicts of interest. Author disclosures are available in the Supporting Information.

## DISCLOSURE

Sophia Wang receives book royalties from APPI and DSMB consultant fees (total less than $2000/year). Andrew J. Saykin receives support from multiple NIH grants (P30 AG010133, P30 AG072976, R01 AG019771, R01 AG057739, U01 AG024904, R01 LM013463, R01 AG068193, T32 AG071444, and U01 AG068057 and U01 AG072177). He has also received support from Avid Radiopharmaceuticals, a subsidiary of Eli Lilly (in kind contribution of PET tracer precursor); Bayer Oncology (Scientific Advisory Board); Eisai (Scientific Advisory Board); Siemens Medical Solutions USA, Inc. (Dementia Advisory Board); Springer‐Nature Publishing (Editorial Office Support as Editor‐in‐Chief, Brain Imaging and Behavior). All other authors report no potential conflicts of interest.

## CONSENT STATEMENT

Informed consent was obtained from all participants at the individual ADRCs.

## Supporting information




**Supporting Information**: alz71467‐sup‐0001‐tableS1‐S5.docx

**Supporting Information**: alz71467‐sup‐0002‐SuppMat.pdf

## References

[1] Loizos M , Neugroschl J , Zhu CW , et al. Adapting Alzheimer's disease and related dementias clinical research evaluations in the age of Covid‐19. Alzheimer Dis Assoc Disord. 2021;35:172‐177. doi:10.1097/WAD.0000000000000455 33901048 PMC8137642

[2] Jacobs DM , Peavy GM , Banks SJ , Gigliotti C , Little EA , Salmon DP . A survey of smartphone and interactive video technology use by participants in Alzheimer's disease research: implications for remote cognitive assessment. Alzheimer's Dement (Amst). 2021;13:e12188. doi:10.1002/dad2.12188 34027018 PMC8132053

[3] Perrin A , Atske S . 7% of Americans don't use the internet. Who are they? Pew Research Center 2021. https://www.pewresearch.org/short‐reads/2021/04/02/7‐of‐americans‐dont‐use‐the‐internet‐who‐are‐they/

[4] Faverio M . Share of those 65 and older who are tech users has grown in the past decade. Pew Research Center 2022. https://www.pewresearch.org/short‐reads/2022/01/13/share‐of‐those‐65‐and‐older‐who‐are‐tech‐users‐has‐grown‐in‐the‐past‐decade/

[5] Perrin A , Anderson M . Tech Adoption climbs among older adults. Pew Research Center 2017. https://www.pewresearch.org/internet/2017/05/17/tech‐adoption‐climbs‐among‐older‐adults/

[6] Chu JN , Kaplan C , Lee JS , Livaudais‐Toman J , Karliner L . Increasing telehealth access to care for older adults during the COVID‐19 pandemic at an academic medical center: video Visits for Elders Project (VVEP). Jt Comm J Qual Patient Saf. 2022;48:173‐179. doi:10.1016/j.jcjq.2021.11.006 35027304 PMC8645266

[7] Perrin A , Atske S . Home broadband adoption, computer ownership vary by race, ethnicity in the U.S. Pew Research Center 2021. https://www.pewresearch.org/short‐reads/2021/07/16/home‐broadband‐adoption‐computer‐ownership‐vary‐by‐race‐ethnicity‐in‐the‐u‐s/

[8] Mitchell UA , Chebli PG , Ruggiero L , Muramatsu N . The digital divide in health‐related technology use: the significance of race/ethnicity. Gerontologist. 2019;59:6‐14. doi:10.1093/geront/gny138 30452660

[9] Mayeda ER , Glymour MM , Quesenberry CP , Whitmer RA . Inequalities in dementia incidence between six racial and ethnic groups over 14 years. Alzheimers Dement. 2016;12:216‐224. doi:10.1016/j.jalz.2015.12.007 26874595 PMC4969071

[10] Rahman M , White EM , Mills C , Thomas KS , Jutkowitz E . Rural‐urban differences in diagnostic incidence and prevalence of Alzheimer's disease and related dementias. Alzheimers Dement. 2021;17:1213‐1230. doi:10.1002/alz.12285 33663019 PMC8277695

[11] Hill CV , Pérez‐Stable EJ , Anderson NA , Bernard MA . The National Institute on Aging Health Disparities Research Framework. Ethn Dis. 2015;25:245‐254. doi:10.18865/ed.25.3.245 26675362 PMC4671408

[12] Nunnerley M , Mattek N , Kaye J , Beattie Z . Preferences of NIA Alzheimer's Disease Research Center participants regarding remote assessment during the COVID‐19 pandemic. Alzheimer's Dement (Amst). 2022;14:e12373. doi:10.1002/dad2.12373 36419636 PMC9677364

[13] Besser L , Kukull W , Knopman DS , et al. Version 3 of the National Alzheimer's Coordinating Center's Uniform Data Set. Alzheimer Dis Assoc Disord. 2018;32:351‐358. doi:10.1097/WAD.0000000000000279 30376508 PMC6249084

[14] Morris JC , Weintraub S , Chui HC , et al. The Uniform Data Set (UDS): clinical and cognitive variables and descriptive data from Alzheimer Disease Centers. Alzheimer Dis Assoc Disord. 2006;20:210‐216. doi:10.1097/01.wad.0000213865.09806.92 17132964

[15] Morris JC . The Clinical Dementia Rating (CDR): current version and scoring rules. Neurology. 1993;43:2412‐2414. doi:10.1212/wnl.43.11.2412-a 8232972

[16] Lopez AB , López G , Hugo M . Hispanics and mobile access to the internet. Pew Research Center 2016. https://www.pewresearch.org/race‐and‐ethnicity/2016/07/20/3‐hispanics‐and‐mobile‐access‐to‐the‐internet/

[17] Aggarwal R , Sidnam‐Mauch E , Neffa‐Creech D , et al. Development of a mobile‐first registry to recruit healthy volunteers and members of underrepresented communities for Alzheimer's disease prevention studies. J Prev Alzheimers Dis. 2023;10:857‐864. 37874108 10.14283/jpad.2023.86 PMC10884078

[18] Suntai Z , Beltran SJ . The intersectional impact of race/ethnicity and sex on access to technology among older adults. Gerontologist. 2023;63:1162‐1171. doi:10.1093/geront/gnac178 36477498 10.1093/geront/gnac178

[19] Mao A , Tam L , Xu A , et al. Barriers to telemedicine video visits for older adults in independent living facilities: mixed methods cross‐sectional needs assessment. JMIR Aging. 2022;5:e34326. doi:10.2196/34326 35438648 10.2196/34326 PMC9066341

[20] Hawley CE , Genovese N , Owsiany MT , et al. Rapid integration of home telehealth visits amidst COVID‐19: what do older adults need to succeed?. J Am Geriatr Soc. 2020;68:2431‐2439. doi:10.1111/jgs.16845 32930391 10.1111/jgs.16845 PMC8554768

[21] Wild KV , Mattek N , Maxwell SA , Dodge HH , Jimison HB , Kaye JA . Computer related self‐efficacy and anxiety in older adults with and without mild cognitive impairment. Alzheimers Dement. 2012;8:544‐552. doi:10.1016/j.jalz.2011.12.008 23102124 10.1016/j.jalz.2011.12.008 PMC3483565

[22] Sixsmith A , Horst BR , Simeonov D , Mihailidis A . Older people's use of digital technology during the COVID‐19 pandemic. Bull Sci Technol Soc. 2022;42:19‐24. doi:10.1177/02704676221094731 38603230 10.1177/02704676221094731 PMC9038938

[23] 2024 Alzheimer's disease facts and figures. Alzheimers Dement 2024;20:3708‐3821. 38689398 10.1002/alz.13809 PMC11095490

