# Supplementary material for: Technology access and preferences for remote assessments at Alzheimer's Disease Research Centers
Source: Alzheimers Dement. 2026 May 11;22(5):e71467. doi: 10.1002/alz.71467 (PMC13160918; doi:10.1002/alz.71467)
Supplement: Supplementary file 1 — Supporting Information: alz71467‐sup‐0001‐tableS1‐S5.docx [file ALZ-22-e71467-s002.docx]

Supplemental Table 1. Participant characteristics stratified across four racial and ethnic groups. CDR = clinical dementia rating scale; yrs = years; MCI = mild cognitive impairment, SD = standard deviation.

|  | *Non-Hispanic White* | | *Hispanic* | | *Non-Hispanic Black* | | *Non-Hispanic Asian & Other race* | |  |
| --- | --- | --- | --- | --- | --- | --- | --- | --- | --- |
| *Variable* | *N* | *Statistic* | *N* | *Statistic* | *N* | *Statistic* | *N* | *Statistic* | *Overall P-value* |
| Age (yrs) at visit, mean ± SD | 3,022 | 73.3 ± 10.1 | 163 | 73.3 ± 10.8 | 500 | 73.2 ± 8.7 | 118 | 73.8 ± 9.5 | 0.952 |
| Female, n (%) | 3,022 | 1,606 (53.1%) | 163 | 109 (66.9%) | 500 | 379 (75.8%) | 118 | 69 (58.5%) | <0.001 |
| Years of Education, mean ± SD | 3,012 | 16.6 ± 2.5 | 163 | 14.2 ± 4.5 | 500 | 15.4 ± 2.5 | 118 | 16.7 ± 2.7 | <0.001 |
| Years of Education, n (%) | 3,022 |  | 163 |  | 500 |  | 118 |  | <0.001 |
| <12 years |  | 14 (0.5%) |  | 29 (17.8%) |  | 9 (1.8%) |  | 1 (0.8%) |  |
| 12+ years |  | 3,008 (99.5%) |  | 134 (82.2%) |  | 491 (98.2%) |  | 117 (99.2%) |  |
| Cognitive status at UDS visit, n (%) | 3,022 |  | 163 |  | 500 |  | 118 |  | <0.001 |
| Normal cognition |  | 1,750 (57.9%) |  | 106 (65.0%) |  | 325 (65.0%) |  | 82 (69.5%) |  |
| Impaired - Not MCI |  | 159 (5.3%) |  | 5 (3.1%) |  | 25 (5.0%) |  | 3 (2.5%) |  |
| MCI |  | 448 (14.8%) |  | 24 (14.7%) |  | 108 (21.6%) |  | 17 (14.4%) |  |
| Dementia |  | 665 (22.0%) |  | 28 (17.2%) |  | 42 (8.4%) |  | 16 (13.6%) |  |
| Global CDR, n (%) | 3,022 |  | 163 |  | 500 |  | 118 |  | <0.001 |
| 0.0 |  | 1,653 (54.7%) |  | 92 (56.4%) |  | 300 (60.0%) |  | 72 (61.0%) |  |
| 0.5 |  | 885 (29.3%) |  | 51 (31.3%) |  | 170 (34.0%) |  | 34 (28.8%) |  |
| ≥1 |  | 484 (16.0%) |  | 20 (12.3%) |  | 30 (6.0%) |  | 12 (10.2%) |  |
| Indicator of first-degree family member with cognitive impairment, n (%) | 2,846 | 1,781 (62.6%) | 148 | 93 (62.8%) | 453 | 238 (52.5%) | 108 | 56 (51.9%) | <0.001 |
| Principal referral source, n (%) | 2,998 |  | 162 |  | 496 |  | 117 |  | <0.001 |
| Non-professional contact |  | 1,115 (37.2%) |  | 55 (34.0%) |  | 211 (42.5%) |  | 52 (44.4%) |  |
| Professional contact |  | 1,338 (44.6%) |  | 71 (43.8%) |  | 146 (29.4%) |  | 43 (36.8%) |  |
| Other referral |  | 545 (18.2%) |  | 36 (22.2%) |  | 139 (28.0%) |  | 22 (18.8%) |  |
| Preference to conduct a study visit, n (%) | 2,944 |  | 160 |  | 482 |  | 117 |  | 0.347 |
| In person |  | 1,280 (43.5%) |  | 73 (45.6%) |  | 195 (40.5%) |  | 52 (44.4%) |  |
| Telephone call |  | 523 (17.8%) |  | 27 (16.9%) |  | 110 (22.8%) |  | 25 (21.4%) |  |
| Video call |  | 530 (18.0%) |  | 27 (16.9%) |  | 78 (16.2%) |  | 15 (12.8%) |  |
| No preference |  | 611 (20.8%) |  | 33 (20.6%) |  | 99 (20.5%) |  | 25 (21.4%) |  |
| Current access to internet via a smartphone, n (%) | 3,022 | 2,076 (68.7%) | 162 | 117 (72.2%) | 498 | 312 (62.7%) | 118 | 88 (74.6%) | 0.013 |
| Current access to internet via a tablet/iPad, n (%) | 3,022 | 1,289 (42.7%) | 162 | 69 (42.6%) | 498 | 189 (38.0%) | 118 | 51 (43.2%) | 0.265 |
| Current access to internet via a laptop, n (%) | 3,022 | 1,838 (60.8%) | 162 | 87 (53.7%) | 498 | 268 (53.8%) | 118 | 74 (62.7%) | 0.009 |
| Current access to internet via a desktop computer, n (%) | 3,022 | 1,280 (42.4%) | 162 | 62 (38.3%) | 498 | 192 (38.6%) | 118 | 45 (38.1%) | 0.269 |
| Uses email to receive and send documents, n (%) | 2,978 | 2,620 (88.0%) | 161 | 116 (72.0%) | 482 | 394 (81.7%) | 118 | 105 (89.0%) | <0.001 |
| Interested in using: Smartphone, n (%) | 3022 | 1,675 (55.4%) | 163 | 102 (62.6%) | 500 | 271 (54.2%) | 118 | 67 (56.8%) | 0.294 |
| Interested in using: Table/iPad, n (%) | 3022 | 1,177 (38.9%) | 163 | 70 (42.9%) | 500 | 192 (38.4%) | 118 | 41 (34.7%) | 0.565 |
| Interested in using: Laptop, n (%) | 3022 | 1,694 (56.1%) | 163 | 72 (44.2%) | 500 | 266 (53.2%) | 118 | 63 (53.4%) | 0.020 |
| Interested in using: Desktop computer, n (%) | 3022 | 1,130 (37.4%) | 163 | 56 (34.4%) | 500 | 186 (37.2%) | 118 | 43 (36.4%) | 0.887 |
| Interested in using: Wearable devices, n (%) | 3022 | 612 (20.3%) | 163 | 28 (17.2%) | 500 | 92 (18.4%) | 118 | 29 (24.6%) | 0.351 |
| Interested in using: Smart home devices, n (%) | 3022 | 258 (8.5%) | 163 | 15 (9.2%) | 500 | 42 (8.4%) | 118 | 11 (9.3%) | 0.979 |

Supplemental table 2. Multinomial logistic regression model using generalized estimating equations with random effect for study site on study visit preferences by racial and ethnic groups. Adjusted for age, sex, education, CDR, and race-education interaction. Reference group = in-person visit. OR = odds ratio; CI = confidence interval; CDR = clinical dementia rating scale; yrs = years.

|  | *Telephone*  *vs. In Person* | | | *Video*  *vs. In Person* | | | *No preference*  *vs. In Person* | | |
| --- | --- | --- | --- | --- | --- | --- | --- | --- | --- |
| *Independent Variable* | *OR* | *95% CI* | *P-value* | *OR* | *95% CI* | *P-value* | *OR* | *95% CI* | *P-value* |
| Age (yrs) at visit | 1.02 | 1.00-1.03 | 0.007 | 0.99 | 0.97-1.00 | 0.135 | 0.99 | 0.97-1.00 | 0.024 |
| Female | 1.40 | 1.14-1.72 | 0.001 | 1.22 | 1.05-1.43 | 0.011 | 1.13 | 0.84-1.52 | 0.418 |
| Years of Education | 0.97 | 0.92-1.02 | 0.271 | 1.08 | 1.04-1.12 | <0.001 | 1.04 | 1.00-1.08 | 0.062 |
| Global CDR |  |  |  |  |  |  |  |  |  |
| 0.0 | 0.63 | 0.26-1.50 | 0.293 | 0.58 | 0.24-1.40 | 0.226 | 1.27 | 0.66-2.43 | 0.473 |
| 0.5 | 0.60 | 0.25-1.44 | 0.253 | 0.59 | 0.29-1.18 | 0.136 | 0.87 | 0.56-1.35 | 0.526 |
| ≥1 | 1.00 | 1.00-1.00 |  | 1.00 | 1.00-1.00 |  | 1.00 | 1.00-1.00 |  |
| Racial/Ethnic Group |  |  |  |  |  |  |  |  |  |
| Hispanic | 0.54 | 0.23-1.26 | 0.154 | 0.36 | 0.01-10.98 | 0.560 | 1.98 | 0.72-5.39 | 0.184 |
| Non-Hispanic Black | 2.16 | 0.75-6.26 | 0.154 | 0.17 | 0.02-1.20 | 0.076 | 1.62 | 0.40-6.60 | 0.503 |
| Non-Hispanic Asian & Other race | 0.57 | 0.05-5.97 | 0.640 | 0.09 | <0.01-1.84 | 0.119 | 0.63 | 0.04-9.15 | 0.734 |
| Non-Hispanic White | 1.00 | 1.00-1.00 |  | 1.00 | 1.00-1.00 |  | 1.00 | 1.00-1.00 |  |
| Education x Racial/Ethnic Group |  |  |  |  |  |  |  |  |  |
| Hispanic | 1.03 | 0.97-1.09 | 0.306 | 1.07 | 0.87-1.31 | 0.510 | 0.96 | 0.88-1.04 | 0.290 |
| Non-Hispanic Black | 0.97 | 0.91-1.03 | 0.325 | 1.12 | 0.99-1.27 | 0.076 | 0.97 | 0.89-1.06 | 0.559 |
| Non-Hispanic Asian & Other race | 1.05 | 0.90-1.21 | 0.549 | 1.13 | 0.96-1.32 | 0.145 | 1.03 | 0.88-1.20 | 0.732 |
| Non-Hispanic White | 1.00 | 1.00-1.00 |  | 1.00 | 1.00-1.00 |  | 1.00 | 1.00-1.00 |  |
|  |  |  |  |  |  |  |  |  |  |

Supplemental table 3. Logistic regression models using generalized estimating equations with random effect for study site on current devices used to access the internet across four racial and ethnic groups. Adjusted for age, sex, education, CDR, and race-education interaction. OR = odds ratio; CI = confidence interval; CDR = clinical dementia rating scale; yrs = years.

|  | *Smartphone* | | | *Tablet* | | | *Laptop* | | | *Desktop* | | |
| --- | --- | --- | --- | --- | --- | --- | --- | --- | --- | --- | --- | --- |
| *Independent Variable* | *OR* | *95% CI* | *P-value* | *OR* | *95% CI* | *P-value* | *OR* | *95% CI* | *P-value* | *OR* | *95% CI* | *P-value* |
| Age (yrs) at visit | 0.93 | 0.92-0.94 | <.001 | 0.98 | 0.97-0.99 | <.001 | 0.95 | 0.94-0.96 | <.001 | 1.00 | 0.99-1.01 | 0.869 |
| Female | 0.83 | 0.73-0.95 | 0.007 | 1.08 | 0.95-1.21 | 0.235 | 0.87 | 0.76-1.01 | 0.061 | 0.81 | 0.74-0.88 | <.001 |
| Years of Education | 1.06 | 1.02-1.10 | 0.004 | 1.03 | 1.00-1.06 | 0.032 | 1.11 | 1.07-1.15 | <.001 | 1.04 | 1.01-1.07 | 0.022 |
| Global CDR - overall |  |  | <.001 |  |  | 0.050 |  |  | 0.005 |  |  | 0.131 |
| 0.0 | 1.48 | 0.93-2.35 | 0.099 | 1.31 | 1.01-1.71 | 0.044 | 1.79 | 1.26-2.54 | 0.001 | 1.31 | 1.00-1.70 | 0.047 |
| 0.5 | 0.99 | 0.67-1.46 | 0.962 | 1.06 | 0.86-1.32 | 0.568 | 1.33 | 1.06-1.68 | 0.014 | 1.18 | 0.91-1.53 | 0.219 |
| ≥1 | 1.00 | 1.00-1.00 |  | 1.00 | 1.00-1.00 |  | 1.00 | 1.00-1.00 |  | 1.00 | 1.00-1.00 |  |
| Racial/Ethnic Group - overall |  |  | 0.245 |  |  | 0.002 |  |  | 0.034 |  |  | <.001 |
| Hispanic | 3.00 | 0.96-9.35 | 0.058 | 0.11 | 0.02-0.52 | 0.006 | 0.10 | 0.01-1.00 | 0.050 | 0.07 | 0.02-0.27 | <.001 |
| Non-Hispanic Black | 0.69 | 0.18-2.56 | 0.574 | 0.55 | 0.20-1.54 | 0.256 | 0.18 | 0.05-0.66 | 0.010 | 0.61 | 0.23-1.63 | 0.326 |
| Non-Hispanic Asian & Other race | 0.62 | 0.06-6.04 | 0.678 | 0.73 | 0.14-3.69 | 0.702 | 0.14 | <0.01-2.06 | 0.150 | 1.62 | 0.08-31.75 | 0.749 |
| Non-Hispanic White | 1.00 | 1.00-1.00 |  | 1.00 | 1.00-1.00 |  | 1.00 | 1.00-1.00 |  | 1.00 | 1.00-1.00 |  |
| Education*Racial/Ethnic Group - overall |  |  | 0.444 |  |  | 0.003 |  |  | 0.045 |  |  | <.001 |
| Hispanic | 0.94 | 0.87-1.02 | 0.122 | 1.16 | 1.05-1.29 | 0.004 | 1.15 | 1.00-1.33 | 0.045 | 1.19 | 1.08-1.30 | <.001 |
| Non-Hispanic Black | 1.01 | 0.93-1.09 | 0.903 | 1.02 | 0.97-1.08 | 0.438 | 1.10 | 1.01-1.20 | 0.021 | 1.03 | 0.97-1.09 | 0.385 |
| Non-Hispanic Asian & Other race | 1.05 | 0.91-1.20 | 0.522 | 1.02 | 0.93-1.12 | 0.704 | 1.13 | 0.95-1.35 | 0.158 | 0.96 | 0.80-1.14 | 0.635 |
| Non-Hispanic White | 1.00 | 1.00-1.00 |  | 1.00 | 1.00-1.00 |  | 1.00 | 1.00-1.00 |  | 1.00 | 1.00-1.00 |  |
|  |  |  |  |  |  |  |  |  |  |  |  |  |

Supplemental Table 4. Logistic regression models using generalized estimating equations with random effect for study site on interest in using devices to complete parts of their study visits across four racial and ethnic groups. Adjusted for age, sex, education, CDR, and race-education interaction. OR = odds ratio; CI = confidence interval; CDR = clinical dementia rating scale; yrs = years.

|  | *Smartphone* | | | *Tablet* | | | *Laptop* | | | *Desktop* | | | *Wearable Device* | | | *Smart Home Device* | | |  |
| --- | --- | --- | --- | --- | --- | --- | --- | --- | --- | --- | --- | --- | --- | --- | --- | --- | --- | --- | --- |
| *Independent Variable* | *OR* | *95% CI* | *P-value* | *OR* | *95% CI* | *P-value* | *OR* | *95% CI* | *P-value* | *OR* | *95% CI* | *P-value* | *OR* | *95% CI* | *P-value* | *OR* | *95% CI* | *P-value* |  |
| Age (yrs) at visit | 0.95 | 0.94-0.97 | <.001 | 0.97 | 0.96-0.98 | <.001 | 0.95 | 0.94-0.96 | <.001 | 0.99 | 0.98-1.00 | 0.132 | 0.96 | 0.95-0.97 | <.001 | 0.96 | 0.95-0.97 | <.001 |  |
| Female | 0.87 | 0.80-0.95 | 0.003 | 1.08 | 0.96-1.20 | 0.196 | 0.86 | 0.73-1.03 | 0.094 | 0.82 | 0.75-0.91 | <.001 | 1.07 | 0.90-1.26 | 0.452 | 0.92 | 0.69-1.21 | 0.541 |  |
| Years of Education | 1.05 | 1.02-1.08 | <.001 | 1.04 | 1.01-1.08 | 0.013 | 1.12 | 1.08-1.17 | <.001 | 1.04 | 1.01-1.06 | 0.002 | 1.07 | 1.04-1.10 | <.001 | 1.08 | 1.04-1.12 | <.001 |  |
| Global CDR - overall |  |  | 0.025 |  |  | 0.071 |  |  | <.001 |  |  | 0.084 |  |  | 0.040 |  |  | 0.870 |  |
| 0.0 | 1.22 | 0.86-1.73 | 0.261 | 1.27 | 1.04-1.56 | 0.022 | 1.84 | 1.39-2.44 | <.001 | 1.34 | 1.03-1.76 | 0.031 | 1.51 | 1.09-2.08 | 0.013 | 1.08 | 0.72-1.60 | 0.720 |  |
| 0.5 | 0.92 | 0.72-1.18 | 0.533 | 1.15 | 0.96-1.37 | 0.124 | 1.30 | 1.08-1.56 | 0.005 | 1.23 | 0.99-1.52 | 0.062 | 1.31 | 0.95-1.81 | 0.098 | 1.12 | 0.73-1.72 | 0.603 |  |
| ≥1 | 1.00 | 1.00-1.00 |  | 1.00 | 1.00-1.00 |  | 1.00 | 1.00-1.00 |  | 1.00 | 1.00-1.00 |  | 1.00 | 1.00-1.00 |  | 1.00 | 1.00-1.00 |  |  |
| Racial/Ethnic Group - overall |  |  | 0.688 |  |  | <.001 |  |  | 0.003 |  |  | <.001 |  |  | 0.322 |  |  | 0.444 |  |
| Hispanic | 2.98 | 0.32-27.67 | 0.337 | 0.16 | 0.02-1.41 | 0.098 | 0.08 | <0.01-0.68 | 0.022 | 0.08 | 0.02-0.30 | <.001 | 0.18 | 0.01-2.29 | 0.186 | 0.24 | 0.02-3.46 | 0.293 |  |
| Non-Hispanic Black | 0.75 | 0.28-2.00 | 0.564 | 0.52 | 0.17-1.61 | 0.255 | 0.24 | 0.06-1.02 | 0.054 | 0.73 | 0.26-2.11 | 0.567 | 0.65 | 0.21-2.03 | 0.461 | 0.40 | 0.07-2.40 | 0.313 |  |
| Non-Hispanic Asian & Other race | 0.66 | 0.08-5.36 | 0.697 | 0.15 | 0.02-1.09 | 0.061 | 0.14 | 0.02-1.26 | 0.080 | 0.67 | 0.04-10.04 | 0.770 | 0.51 | 0.07-3.66 | 0.501 | 1.77 | 0.19-16.84 | 0.619 |  |
| Non-Hispanic White | 1.00 | 1.00-1.00 |  | 1.00 | 1.00-1.00 |  | 1.00 | 1.00-1.00 |  | 1.00 | 1.00-1.00 |  | 1.00 | 1.00-1.00 |  | 1.00 | 1.00-1.00 |  |  |
| Education x Racial/Ethnic Group - overall |  |  | 0.795 |  |  | <.001 |  |  | 0.005 |  |  | 0.010 |  |  | 0.294 |  |  | 0.562 |  |
| Hispanic | 0.95 | 0.83-1.08 | 0.415 | 1.15 | 0.99-1.33 | 0.066 | 1.16 | 1.00-1.34 | 0.052 | 1.17 | 1.05-1.29 | 0.003 | 1.09 | 0.93-1.29 | 0.280 | 1.08 | 0.91-1.27 | 0.370 |  |
| Non-Hispanic Black | 1.01 | 0.95-1.08 | 0.685 | 1.03 | 0.96-1.11 | 0.389 | 1.09 | 1.00-1.19 | 0.050 | 1.02 | 0.96-1.08 | 0.580 | 1.01 | 0.94-1.08 | 0.793 | 1.06 | 0.94-1.19 | 0.336 |  |
| Non-Hispanic Asian & Other race | 1.02 | 0.90-1.16 | 0.747 | 1.10 | 0.97-1.25 | 0.138 | 1.11 | 0.98-1.27 | 0.106 | 1.02 | 0.87-1.18 | 0.838 | 1.06 | 0.93-1.19 | 0.382 | 0.97 | 0.84-1.11 | 0.632 |  |
| Non-Hispanic White | 1.00 | 1.00-1.00 |  | 1.00 | 1.00-1.00 |  | 1.00 | 1.00-1.00 |  | 1.00 | 1.00-1.00 |  | 1.00 | 1.00-1.00 |  | 1.00 | 1.00-1.00 |  |  |
|  |  |  |  |  |  |  |  |  |  |  |  |  |  |  |  |  |  |  |  |

Supplemental Table 5. Demographics of NACC participants based on CTAS administration. Group 1: Participants who completed CTAS; Group 2: Participants who had a visit on/after 7/2/2020 through 4/23/2023 but not did not complete CTAS at sites that administered CTAS; Group 3: Participants who had a visit on/after 7/2/2020 through 4/23/2023 but not did not complete CTAS at sites that did not administer CTAS; Group 4: Participants with visits 7/2/2017-7/1/2020 but no visits afterwards and did not complete CTAS regardless of site. CTAS = Covid Technology Access Survey; CDR = clinical dementia rating scale; SD = standard deviation.

|  | *1. Participants who completed CTAS* | | *2. No CTAS - participating site* | | *3. No CTAS - non-participating site* | | *4. Only visits prior to CTAS* | |  |
| --- | --- | --- | --- | --- | --- | --- | --- | --- | --- |
| *Characteristic* | *N* | *Statistic* | *N* | *Statistic* | *N* | *Statistic* | *N* | *Statistic* | *Overall P-value* |
| Died before 7/2/2020, n (%) | 3,803 | 0 (0.0%) | 5,194 | 0 (0.0%) | 6,523 | 0 (0.0%) | 6,139 | 1,165 (19.0%) | <0.001 |
| Age on July 2, 2020, mean ± SD | 3,803 | 72.8 ± 9.9 | 5,194 | 71.3 ± 11.1 | 6,523 | 72.2 ± 10.1 | 6,139 | 76.9 ± 11.8 | <0.001 |
| Female, n (%) | 3,803 | 2,163 (56.9%) | 5,194 | 2,968 (57.1%) | 6,523 | 3,943 (60.4%) | 6,139 | 3,488 (56.8%) | <0.001 |
| Years of Education, mean ± SD | 3,793 | 16.3 ± 2.7 | 5,157 | 16.0 ± 3.0 | 6,504 | 16.0 ± 2.8 | 6,087 | 15.4 ± 3.3 | <0.001 |
| Years of Education, n (%) | 3,793 |  | 5,157 |  | 6,504 |  | 6,087 |  | <0.001 |
| <12 years |  | 53 (1.4%) |  | 171 (3.3%) |  | 202 (3.1%) |  | 332 (5.5%) |  |
| 12+ years |  | 3,740 (98.6%) |  | 4,986 (96.7%) |  | 6,302 (96.9%) |  | 5,755 (94.5%) |  |
| Global CDR, n (%) | 3,803 |  | 5,194 |  | 6,523 |  | 6,139 |  | <0.001 |
| 0.0 |  | 2,117 (55.7%) |  | 2,443 (47.0%) |  | 3,416 (52.4%) |  | 2,023 (33.0%) |  |
| 0.5 |  | 1,140 (30.0%) |  | 1,498 (28.8%) |  | 1,996 (30.6%) |  | 1,839 (30.0%) |  |
| ≥1 |  | 546 (14.4%) |  | 1,253 (24.1%) |  | 1,111 (17.0%) |  | 2,277 (37.1%) |  |
| Racial/Ethnic group, n (%) | 3,803 |  | 5,194 |  | 6,523 |  | 6,139 |  | <0.001 |
| Non-Hispanic White |  | 3,022 (79.5%) |  | 3,673 (70.7%) |  | 4,489 (68.8%) |  | 4,519 (73.6%) |  |
| Hispanic |  | 163 (4.3%) |  | 448 (8.6%) |  | 751 (11.5%) |  | 606 (9.9%) |  |
| Non-Hispanic Black |  | 500 (13.1%) |  | 765 (14.7%) |  | 978 (15.0%) |  | 793 (12.9%) |  |
| Non-Hispanic Asian & Other race |  | 118 (3.1%) |  | 308 (5.9%) |  | 305 (4.7%) |  | 221 (3.6%) |  |
